# A microRNA-dependent circuit controlling p63/p73 homeostasis: p53 family cross-talk meets therapeutic opportunity

**DOI:** 10.18632/oncotarget.244

**Published:** 2011-03-23

**Authors:** Benjamin Ory, Leif W. Ellisen

**Affiliations:** Massachusetts General Hospital Cancer Center and Harvard Medical School, Boston, MA 02114, USA

**Keywords:** p63, p73, microRNA, apoptosis, cisplatin, squamous cell carcinoma, triple-negative breast cancer, chemosensitivity

## Abstract

The p53 family transcription factors p53, p63 and p73 make diverse contributions in development and cancer. Mutation or deletion of p53 is observed in the majority of human cancers. In contrast, p63 and p73 are not lost in cancer but mediate distinct genetic roles in normal and tumor-specific contexts: p73 promotes genome stability and mediates chemosensitivity, while p63 largely lacks these p53-like functions and instead promotes proliferation and cell survival. We recently uncovered a mechanism which maintains p63/p73 homeostasis within the epithelium through direct transcriptional regulation of microRNAs (miRs). We discovered that several of the top p63-regulated miRs target p73 for inhibition, including miR-193a-5p, a direct p63/p73 transcriptional target which is repressed by p63 and activated by p73 both *in vitro* and *in vivo*. The resulting feed-forward circuit involving p63, miR-193a-5p and p73 controls p73 levels, cell viability and DNA damage susceptibility in certain cancers including squamous cell carcinoma. Here, we discuss the evolutionary implications of this regulatory circuit, which may point to a general mechanism of miR-mediated cross-talk within transcription factor gene families. Additionally, we suggest that inducible chemoresistance mediated by this miR-dependent mechanism might be an attractive target for therapeutic intervention.

## THE P53 FAMILY: COLLABORATION AND COMPETITION IN EPITHELIAL CANCERS

The p53 gene is the prototypical human tumor suppressor and is mutated or lost in the majority of human cancers. Loss of p53 transcription factor function in cancer reflects the diverse contribution of this protein to the DNA damage response, cell cycle regulation, cell survival and many other functions [1]. Two p53-related genes, p63 and p73, are expressed in mammals, but unlike p53 neither of these genes exhibits frequent somatic mutation in cancer [2, 3]. Both p63 and p73 are expressed as two predominant isoform classes resulting from alternative promoter usage: the TAp63/TAp73 isoforms contain an N-terminal transactivation domain and most resemble p53, while the △Np63/△Np73 isoforms exhibit a truncated N-terminus. Additional isoforms of p63 and p73 are generated through alternative C-terminal mRNA splicing [2, 4]. While TAp63/TAp73 isoforms mediate predominantly transcriptional activation, △Np63/△Np73 isoforms function as transcriptional activators and repressors of distinct sets of transcriptional target genes [3]. Genetic and biochemical studies have identified p53-like functions for p73 in the maintenance of genomic integrity and regulation of apoptosis [5, 6]. In contrast, p63 functions to maintain cellular regenerative proliferation and survival of stratified epithelia [7, 8].

Both p63 and p73 exhibit isoform-specific expression and functions in human cancer [3]. In squamous cell carcinoma (SCC) and certain breast cancers the predominant p63 isoform is △Np63α, which is closely linked to cell survival and adhesion signaling, while in these same tumors pro-apoptotic TAp73 isoforms predominate [9, 10]. Multiple functional interactions occur between these isoforms and are important for tumor maintenance. These include direct physical interaction between p63 and p73 through their homologous oligomerization domains, and regulation of shared transcriptional target genes through direct promoter binding mediated via highly homologous DNA binding domains [4, 11]. In SCC and some triple-negative breast cancers (TNBC, so-called because they lack expression of estrogen receptor, progesterone receptor, and amplified HER2), tumor cell survival depends on the ability of △Np63α to physically associate with TAp73 and thereby abrogate p73-dependent apoptosis [11, 12]. △Np63α also binds directly in a repressive complex at p73-regulated pro-apoptotic gene promoters, providing an additional mode of functional p73 suppression [13]. The therapeutic relevance of these findings is evidenced by the ability of cisplatin chemotherapy, a mainstay of SCC treatment and an emerging agent for TNBC therapy, to target △Np63 for degradation and TAp73 for phosphorylation, thereby activating the p73-dependent pro-apoptotic program [12, 14]. Based on these findings, studying mechanisms which regulate p63/p73 expression and function in these tumors may uncover new therapeutic opportunities.

## MIR-DEPENDENT REGULATION AND THE P53 FAMILY

MicroRNAs (miRs) are small non-coding RNAs that function as post-transcriptional regulators of gene and protein expression. Several miRs have recently been linked to the p53 family. In particular, miR-34a is a direct transcriptional target of p53 which contributes to p53-dependent functions through interaction with p53-regulated mRNAs [15-17]. Additionally, multiple miRs have now been shown to target p53 itself for inhibition, suggesting these miRs may function as oncogenes [18, 19]. TAp63 isoforms function as metastasis suppressors in certain cancers in part through regulation of a miR-dependent program [20], and at least one miR has been identified to control p63 itself and to modulate its developmental role [21]. As evidenced by these examples and as discussed in more detail below, an emerging consensus is that miRs are particularly prominent within regulatory circuits controlling transcription factor functions. Although we are only beginning to uncover their complexity, such circuits may be particularly important within transcription factor gene families.

## A MIR-MEDIATED MECHANISM FOR CROSS-TALK WITHIN THE P53 FAMILY

In order to identify p63-regulated miRs we performed global miR expression profiling following p63 ablation in human squamous cell carcinoma (SCC) cells, which express high levels of endogenous △Np63α. Surprisingly, we observed that three of the top ten most highly-regulated miRs, miR-193a-5p, miR-602, and miR-765, were predicted to target the p73 3'UTR [14]. Each of these miRs was induced following p63 knockdown, suggesting a miR-dependent mechanism for p63 to activate p73. We initially validated this proposed model by demonstrating that p63 controlled gene expression via the p73 3'UTR in a manner dependent upon Drosha, an RNase III-type endonuclease required for miR nuclear processing. We next focused on one of these miRs, miR-193a-5p (designated miR-193* in mouse; hereafter both human and mouse are referred to as miR-193a). We confirmed direct regulation of the p73 3'UTR by a transfected miR-193a mimic, using UTR constructs in which we engineered mutations in the predicted seed binding sequences and showing that these abolished miR-dependent regulation. In order to establish p73 regulation by endogenous miR-193a we introduced a miR-193a antagomir (miR inhibitor), which also showed the expected UTR-dependent regulation of p73 dependent upon the specific miR-193a seed binding sequences [14].

**Figure 1:**
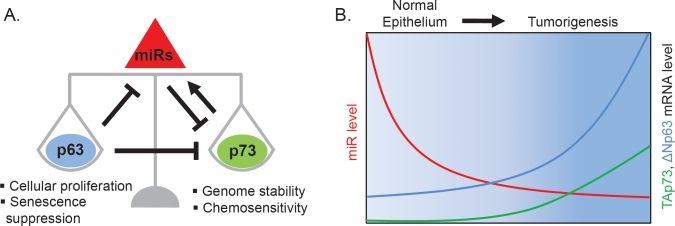
A miR-mediated negative feed-forward loop maintains p63/p73 homeostasis in the epithelium **A.** Proposed miR-mediated feed-forward circuit. P63 is a transcriptional repressor of miRs that target p73 for inhibition. One of these miRs, miR-193a, is a direct transcriptional target repressed by p63 and activated by p73. P63 also inhibits p73 function by direct physical interaction and by binding to shared promoter elements. This p63/p73 circuit is unique in involving two transcription factors which are members of a conserved gene family. Additionally, it is remarkable for implicating three levels of direct regulation: transcriptional regulation by p63 and p73 of the miR; post-transcriptional regulation by the miR of p73, and post-translational regulation by p63 of p73 activity. **B.** Schematic expression levels of p63, p73 and miR-193a in basal cells of stratified squamous epithelium (normal) and squamous cell carcinoma (tumor). Both p63 and p73 are up-regulated in tumors relative to normal cells. Increased p63 expression in tumors mediates miR-193a repression, which in turn contributes to increased p73 mRNA and thereby maintains a balanced p63/p73 ratio. Disruption of the network by miR inhibition increases p73 activity, leading to impaired cellular viability and enhanced chemosensitivity.

We were then interested to know whether regulation of miR-193a by △Np63α was a direct transcriptional effect. We therefore used chromatin immunoprecipitation (ChIP) to map a p63 binding site with the miR-193a locus, and we showed using reporter assays that the canonical p53 family binding sequence within this p63-bound region was required for p63 dependent suppression of this miR. Remarkably, we also observed direct binding of p73, as well as TAp73-dependent regulation of miR-193a following cisplatin chemotherapy treatment, which is known to induce △Np63α degradation and TAp73 activation [14]. Collectively, these findings suggested a feed-forward loop whereby △Np63α expression would suppress miR-193a and thereby increase TAp73 levels, while TAp73 would be involved in negative feedback regulation via its own 3'UTR and miR-193a (Figure 1). These predictions were all experimentally validated. Furthermore, we provided evidence for the validity of this regulatory mechanism in primary SCC specimens, which show variable levels of △Np63α overexpression, by demonstrating a significant inverse correlation between △Np63 and miR-193a levels, and a positive correlation between △Np63 and TAp73 levels [14].

## INDUCIBLE CHEMORESISTANCE THROUGH P63/P73 FEED-FORWARD REGULATION

Given that this miR-dependent circuit converged on regulation of p73, we next tested the contribution of miR-193a in a key physiologic context for p73 function: the response to cisplatin, which as noted above is both an inhibitor of △Np63α and a specific activator of TAp73-dependent transcription and cell death [12, 22]. As noted above, miR-193a was induced by TAp73 in response to cisplatin, and consistent with our proposed model we observed that a miR-193a antagomir substantially increased chemosensitivity in response to cisplatin. Moreover, we found that this effect was specifically attributable to regulation of TAp73. Thus, the difference in chemosensitivity observed in control versus antagomir-treated cells was correlated with induction of TAp73 pro-apoptotic transcriptional target genes, and this difference was abolished when TAp73 knockdown was performed prior to cisplatin treatment [14]. Taken together, these findings argue that induction of miR-193a through the p63 and p73-dependent effects of cisplatin limits p73-dependent chemosensitivity through direct feedback inhibition.

To determine whether these findings could be both generalized and validated *in vivo* we tested the contribution of miR-193a to chemosensitivity in a mouse model of SCC. This model recapitulates the features of human SCC, including high-level p63 expression, squamous differentiation, and metastasis to local lymph nodes [14]. Primary SCC tumors were disaggregated, then re-implanted into multiple mice in the presence of a miR-193a antagomir or control, followed by treatment with cisplatin or vehicle. Notably, we observed that inhibition of miR-193a alone was sufficient to inhibit tumor growth, in keeping with its ability to potentiate the pro-apoptotic activity of TAp73. Most importantly, however, miR-193a inhibition had a dramatic impact on chemosensitivity to cisplatin. Indeed, a cisplatin dose that alone had no significant effect on tumor progression in control antagomir-treated tumors nevertheless completely abolished tumor growth in miR-193a antagomir-treated tumors [14]. These experiments therefore provide tantalizing proof-of-principle for targeting miR expression as a means to enhance chemosensitivity in SCC and potentially other tumors, including TNBC, which express p63 and p73.

## POTENTIAL PHYSIOLOGIC AND EVOLUTIONARY ROLE OF P63/P73 FEED-FORWARD REGULATION

The endogenous regulatory circuit we have identified involves a negative feed-forward loop from p63 to p73 (Figure). While feed-forward transcription factor regulatory networks involving miRs are proposed to be a recurrent motif in mammalian cells [23], to our knowledge only one example, involving c-Myc and E2F1, has been fully experimentally validated [24, 25]. The p63/p73 circuit is unique in involving two transcription factors which are members of a conserved gene family. This circuit is also noteworthy as the first example of such a regulatory motif implicating three levels of direct regulation: transcriptional regulation of the miR by p63 and p73; post-transcriptional regulation of p73 by the miR, and post-translational regulation of p73 activity by p63 through both direct protein interaction and competition at shared promoter elements [9, 11, 12, 26]. The rationale for such complex circuitry has been proposed to involve the dampening of random fluctuations in activation/expression of the involved transcription factors, which thereby prevents inappropriate state switching (e.g. from proliferation to growth arrest or cell death) [23]. Whereas the c-Myc/E2F1 loop is thought to maintain stable expression of E2F1, we provide evidence that the p63/p73 circuit by virtue of its distinct mechanism serves to maintain balanced co-expression of these two factors. Clearly, an imbalance could produce disastrous consequences, given the essential and often opposing roles of p63 and p73 in cellular proliferation and survival (△Np63α), and apoptosis and tumor suppression (TAp73) [5, 27]. Confirming the importance of this miR-dependent homeostatic mechanism, we found that cell viability is compromised when endogenous miR-193a is inhibited, and we confirmed that this is a TAp73-dependent effect. Furthermore, we show that the p63/p73-dependent apoptotic response to chemotherapy is dramatically perturbed in the absence of miR-193a, leading to enhanced cytotoxicity both in vitro and in vivo.

While our findings provide a unique example of a miR-mediated regulatory circuit within a transcription factor gene family, it is conceivable that such miR-dependent mechanisms for feedback and feed-forward regulation may modulate the function of other such families. For example, transcriptional co-regulation of common set of miRs by different members of a transcription factor gene family might be relatively common, while feedback regulation to one of the involved factors might facilitate functional divergence among closely-related family members during evolution. Indeed, the stability provided by miR-mediated circuits has been proposed to contribute to “evolvability” by buffering the phenotypic consequences of genetic variation [28]. In this regard it is of note that the more homologous family members p63 and p73 participate in the miR-193a regulatory circuit, while p53, which is more distant in both in its sequence and function, is not a direct participant. Additional examples of miR-mediated network motifs involving a single transcription factor family will no doubt be uncovered, and they may provide new insights into the contribution of non-coding RNAs to developmental homeostasis.

## THERAPEUTIC IMPLICATIONS OF MIR-DEPENDENT P63/P73 REGULATION

The critical role of p53 as a tumor suppressor has focused attention on various means to target the p53 pathway as a therapeutic strategy [29]. Unfortunately, the loss or mutation of p53 observed in many cancers presents substantial challenges to efforts aimed at activating p53 itself in tumors. Furthermore, tumors harboring p53 loss of function are in general associated with treatment resistance and a relatively poorer prognosis. We and others have observed that a subset of tumors exhibiting mutational activation of p53 retain or indeed up-regulate pro-apoptotic isoforms of p73 [9, 30]. This tumor-specific TAp73 expression may represent a response to ongoing and/or unrepaired spontaneous DNA damage in tumor cells. Despite TAp73 expression, however, tumors utilize a variety of mechanisms to suppress TAp73 activity and thereby avoid its lethal consequences [3]. Nevertheless, TAp73 can be activated by chemotherapy and other DNA damaging agents, triggering a TAp73-dependent apoptotic response. The ability of certain DNA damaging agents including cisplatin to activate TAp73 may therefore explain the correlation of p73 levels with chemotherapy response in a variety of tumor-specific contexts [31, 32].

Given these observations, our discovery that a miR-dependent mechanism controls p73 levels and activity in certain cancers provides a new means to target the TAp73-dependent apoptotic response for tumor-specific killing. The finding that this miR participates in a feed-forward regulatory loop with △Np63α suggests particular relevance for this mechanism in epithelial tumors which co-express these factors, including SCC and TNBC. Specifically, our data suggest that TAp73-dependent induction of miR-193a following chemotherapy functions as a mechanism of inducible chemoresistance by limiting the TAp73-mediated DNA damage response. In keeping with this hypothesis, we demonstrate using our orthotopic tumor model that a completely ineffective chemotherapy dose can completely block tumor progression when combined with miR-193a inhibition. Importantly, complementary in vitro experiments show that the potentiation of chemosensitivity following miR inhibition is a TAp73-dependent effect. Cisplatin was the chemotherapy agent of choice for these experiments, owing both to its specific activation of TAp73 and to its use as a mainstay of SCC therapy and a potentially important agent for treatment of TNBC [31, 33]. Thus, targeting miR-193a for chemosensitization may represent an attractive future treatment strategy.

Perhaps even more exciting from a therapeutic standpoint, our findings suggest that TAp73 activation through miR inhibition may be associated with a therapeutic effect even in the absence of DNA damaging chemotherapy. This observation is in line with a body of work by our group and others demonstrating that activation of TAp73 may represent a common stress response mechanism, particularly in p53-mutant tumors. For example, recent work suggests that in addition to DNA damage, TAp73 is activated in response to growth factor withdrawal and metabolic stress [34, 35]. Given that these pathways are already being selectively targeted in cancer therapy, the potential to enhance the lethal response to such therapies through concurrent miR suppression leading to TAp73 hyper-activation is quite appealing.

A conceptually similar combination might be envisioned between miR-193a inhibition and the new generation of targeted therapies that aim to disable DNA repair itself. The prototype of these drugs are inhibitors of poly ADP(ribose) polymerase (PARP), enzymes required for single-strand break and base-excision repair [36]. Combinations of PARP inhibitors with DNA damaging chemotherapy are already showing early promise in the treatment of TNBC [37, 38]. Activation of TAp73 may be involved in the response to unrepaired DNA damage in at least a subset of these tumors. Additionally, PARP inhibitors are known to disable repair of spontaneous DNA damage in the absence of chemotherapy, an effect which is likely to trigger TAp73 activation. Therefore, it is attractive to speculate that the combination of a PARP inhibitor with miR-193a inhibition in these tumors might be sufficient to induce a TAp73-dependent therapeutic response in the absence of chemotherapy. In theory, such a combination might be more tumor-selective and less toxic than a PARP inhibitor/chemotherapy combination, owing in part to high levels of TAp73 expressed in some tumors relative to normal cells.

## CONCLUSIONS

The prominent role of miRs as regulators of gene expression, and their deregulation in human cancer provide hope that these molecules may serve as important tools for cancer detection, diagnosis and prognostication. Our work demonstrates in addition how uncovering the detailed functional contribution of miRs in cancer could open the door to a new class of miR-targeted cancer therapies. We have revealed a central role for the p63-regulated miR-193a in maintaining p63/p73 homeostasis within the epithelium. Selective targeting of this miR enhances chemosensitivity, and may have future applications in modulating the response to targeted therapeutics in selected cancer subtypes. Understanding the complex regulatory circuits in which miRs function, as well as their tumor-specific context, will be essential for realizing the promise of miRs in cancer diagnosis and therapy.
